# Seasonal influence on the bathymetric distribution of an endangered fish within a marine protected area

**DOI:** 10.1038/s41598-021-92633-x

**Published:** 2021-06-25

**Authors:** A. Brazo, R. Marques, M. Zimmermann, E. Aspillaga, B. Hereu, G. Saragoni, A. Mercière, R. Crec’Hriou, M. Mercader, M. Verdoit-Jarraya, F. Cadène, P. Lenfant

**Affiliations:** 1 Centre de Formation et de Recherche sur les Environnements Méditerranéens, UMR 5110, Université de Perpignan Via Domitia, 66860 Perpignan, France; 2 CNRS, UMR 5110, Centre de Formation et de Recherche SUR LES Environnements Méditerranéens, 66860 Perpignan, France; 3 Centre de Recherche sur les Ecosystèmes Marins – Plateforme Intervention et Expertise en Environnement Marin (CREM-IEEM), impasse du solarium, 66420 Le Barcares, France; 4 Department of Marine Ecology, Institut Mediterrani d’Estudis Avançats, IMEDEA (CSIC-UIB), C/Miquel Marquès 21, 07190 Esporles, Balearic Islands Spain; 5 Department of Evolutionary Biology, Ecology and Environmental Sciences, Institut de Recerca de La Biodiversitat (IRBIO), University of Barcelona (UB), Av. Diagonal 643, 08028 Barcelona, Spain; 6 PSL Université - EPHE-UPVD-CNRS, USR 3278 CRIOBE (Papetoai), Perpignan, France; 7 Station Biologique CNRS-Sorbonne Université - Service Observation, Place Georges Teissier CS90074, 29688 Roscoff, France; 8 Marine Eco-Evo-Devo Unit, Okinawa Institute of Science and Technology, Onna-son, Okinawa, Japan; 9 Réserve Naturelle Marine de Cerbère Banyuls, 5 rue Roger David, 66650 Banyuls-sur-Mer, France; 10 German Center for Marine Biodiversity Research (DZMB), Senckenberg am Meer, Martin-Luther-King Platz 3, 20146 Hamburg, Germany

**Keywords:** Marine biology, Behavioural ecology, Conservation biology, Behavioural ecology, Conservation biology

## Abstract

The spatio-temporal variability of fish distribution is important to better manage and protect the populations of endangered species. In this sense, the vertical movements of a vulnerable and protected species, *Sciaena umbra*, were assessed in a marine protected area (the *Réserve Naturelle Marine de Cerbère-Banyuls*, south of France) to study the variability of their bathymetric distribution at different time scales. Twenty adults were marked with acoustic transmitters and acoustically monitored over 2.5 years. This revealed that some individuals remained at shallow waters (< 8 m) all year round, while others presented vertical segregation at deeper waters during the cold months (mean depth of 22.5 ± 0.04 m) and all aggregated in shallow waters during the warm months. The brown meagre was more active during the night, except in June and July when peaks of activity were observed at dusk. These patterns are likely associated with foraging and reproductive behavior during the cold and warm periods, respectively, and likely regulated by water temperature and the depth of the thermocline. Here, we provide valuable information on when and where in the water column critical periods of *S. umbra* life cycle are expected to occur, which should be considered in management and protection plans.

## Introduction

The Mediterranean Basin is a biodiversity hotspot with conservation priorities due to its high number of endemic and endangered species, but also due to the anthropogenic pressure that it has suffered for centuries^1^. According to IUCN Red List of Threatened Species (https://www.iucnredlist.org/regions/mediterranean), over 20% of the studied Mediterranean species are endangered, which stresses the importance of marine protected areas (MPAs) for the conservation of marine biodiversity in this region. MPAs are among the most applied and effective marine conservation strategies^2^. Their main purpose is often the protection of habitats that represent important areas for focal species (usually endangered), such as their residence, nursery, spawning or feeding areas, to ultimately protect the local biodiversity^3^. However, one of the major challenges in conservation biology is to understand when and where each critical phase of the targeted species life cycle occurs (e.g. feeding activity, spawning behavior, and site fidelity) to facilitate the development of effective management strategies through MPAs.

Critical periods on a species life cycle might be recognized by specific behaviors or movements of individuals within the local population in terms of habitat use in space and time. One of the best examples is the migration of most grouper species to specific spawning areas all around the world^4–6^. These critical periods are usually synchronized with environmental conditions, as it has been observed for many living organisms such as birds^7^, plankton^8^, and fish^9^. Mechanisms that trigger life cycle events seem to be regulated by complex physiological reactions, like hormone secretion or gene expression^10–12^, which are in turn linked to some cyclical and predictable environmental factors (e.g. seasons, semi-lunar and circadian cycles)^13^. These seasonal, circannual and circadian biological rhythms are not only immediate responses to the environmental variations but constitute some anticipation of these events driven by endogenous factors, corresponding to the internal biological clock inscribed in their genes^7^. The anticipation of seasonal and daily changes constitutes one of the foundations for a successful adaptation of the organisms to the environmental conditions of a given geographic site and play a major complementary role in the success of an ecological function. This is the case of the reproduction period of some temperate fish, which is seasonally controlled by neuroendocrine regulation in relation to the photoperiodic cycle^10^.

In addition to the consistent seasonal and daily patterns of some environmental factors, the marine organisms are also under the effect of fluctuating factors, such as seawater temperature^14^ and salinity^15^, which might vary with wind conditions over short periods. Temperature is one of the most important factors influencing all biological activities^16^ and, in particular, ectothermic organisms, such as fish, can tolerate a very narrow range of temperature. Thus, these organisms must search areas where the temperature is within their thermal optimum to ensure their best physiological performance during growth, reproduction, foraging activity^17–19^ and, more globally, to attain better fitness^20^. The vertical distribution of temperature in aquatic ecosystems will, therefore, control the distribution of the organisms through the water column, which is particularly important for ectothermic fish^21^.

The brown meagre (*Sciaena umbra*) is very vulnerable to fishing (spearfishing, recreational and commercial), due to its high commercial value, its highly accessible habitats, its gregarious character, and its peace behavior, which make it an easy target^22–24^. Heavy exploitation caused such a decrease in *S. umbra* populations that the species is currently classified as endangered in the Mediterranean region^25–27^. MPAs have proven to be beneficial to this species, effectively contributing to increase its abundance and biomass within the protected areas^28,29^. Due to the persistent decrease in the number of individuals and scarcity of brown meagre on the French Mediterranean coasts, a moratorium was approved, which bans the capture of *S. umbra* over the entire French Mediterranean coast until 2023^30^. However, further improvement in the knowledge of this little known species is required to sustain the persistence of this protection measure^31^. Several studies on the biology of the brown meagre have been made in the Eastern Mediterranean^32–34^, but key aspects are still unknown, such as their spatial and temporal movement patterns, which are imperative for proper management of the species^35^. Temperature is recognized as one of the most important factors affecting the maturation of the gonads of the brown meagre, determiningly the efficiency of the species reproduction^18^ and exceeding warmer waters might, therefore, influence this success^17^. Therefore, considering that this species has high site fidelity^36^, vertical movements at specific periods of its life-cycle might be expected, as observed for other species (e.g. Furukawa et al. 2014; Aspillaga et al. 2017). Identifying such movements and the potential association with temperature is of high importance, not only to improve the effectiveness of protection measures but also to understand the potential impact of climate change on the spatial distribution of this species and its reproductive activity.

The development of acoustic telemetry, including small acoustic transmitters, has enabled researchers to conduct studies on fish movements to model the spatial and temporal activity^37–39^, to determine site fidelity^40,41^ and to specify habitat use^37,42,43^. Acoustic telemetry offers many advantages for monitoring fish movements compared to traditional methods such as mark-recapture or visual observation^44,45^. Passive tracking techniques that use networks of acoustic receivers allow to continuously monitor the movements of several individuals at high spatial and temporal resolutions and without human interference^44,45^. Indeed, several studies have successfully used acoustic telemetry to elucidate the movement patterns of fish in MPAs^39,46–49^, including in the Mediterranean Sea^37,43,50–52^. However, very few studies have been carried out on the movements of the brown meagre^36,53^ and those were performed on few individuals and over short periods of less than 1 year. Furthermore, acoustic telemetry studies are predominantly focused on their spatial distribution and fish vertical movements are typically ignored. This potentially masks important behavior that might help us understand fish ecology and contribute to their successful protection.

In this study, although we recognize that further spatial investigations should be performed, we focused on the little known bathymetric distribution of endangered species at different time scales and over more than 2 years of survey, in order to better understand when and where in the water column critical periods of the brown meagre life cycle occur. We used acoustic telemetry to uncover seasonal and daily variations of their vertical movements within an MPA and highlight the environmental factors influencing these patterns. The impact of anthropogenic activities on the behavior of this fish was also tested through the influence of the MPA protection level. Here, we provide useful information to managers to find appropriate and more effective protection measures for this endangered species, which might be potentially implemented at national and European scales.

## Material and methods

### Study site

**Figure 1:**
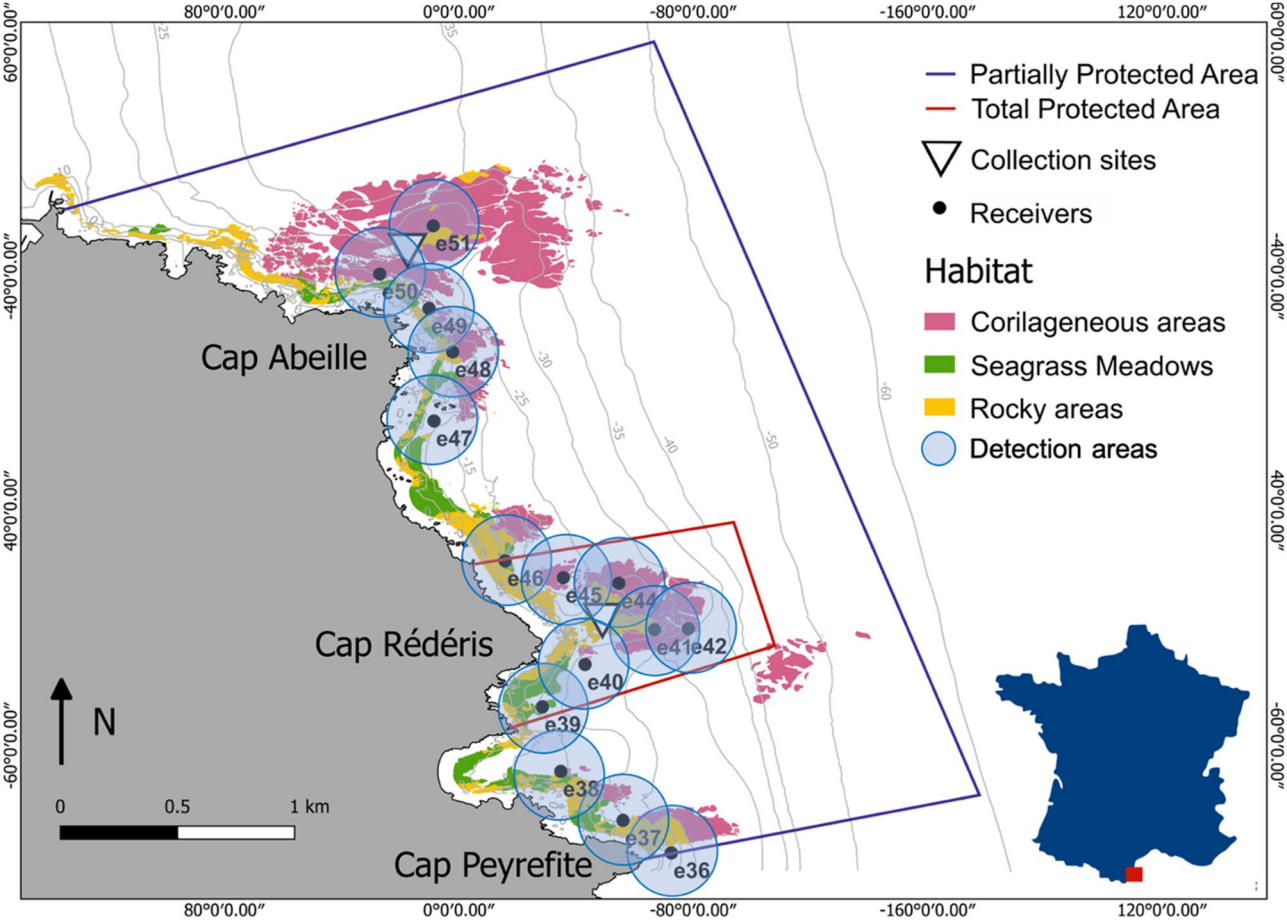
Study area and the acoustic receiver array within the RNMCB (Réserve Naturelle Marine de Cerbère-Banyuls), indicating the areas with different protection levels, the two collection sites (Close to Cap Abeille and Cap Rédéris), the receivers and their detection range (200 m) and the distribution of the main habitats types. Grey lines are bathymetric contours. The basic cartographic map and bathymetric data were provided by RNMCB.

The vertical distribution of the brown meagre (*Sciaena umbra*) was studied within an MPA (the *Réserve Naturelle Marine de Cerbère-Banyuls*, RNMCB), between June 2016 and December 2018. Created in 1974, this MPA is located in the North-Western Mediterranean Sea (Fig. 1). It covers 6.5 km of coastline and up to 1.5 nautical miles offshore, for a total area of 650 ha^54^. The RNMCB includes two areas with different levels of protection: the Totally Protected Area (TPA), with 65 hectares nearby Cap Rédéris, whereonly recreational navigation, surface swimming, and scientific diving are authorized; and the Partially Protected Area (PPA), located in the surrounding zone (585 hectares), where humanactivities are allowed under regulation (e.g. boat circulation, fishing and diving). This protected area has a great diversity of habitats (e.g. rock and boulder bottoms, coralligenous outcrops, and seagrass meadows), providing a suitable environment for the brown meagre^31,54^. Indeed, the RNMCB harbors several individuals of this species, with an apparent increasing trend in abundance (from 175 in 2001 to 321 in 2017, Cadéne, personal communication) and, therefore, it represents an ideal study site to investigate the ecology of the species.

### Fish sampling, tagging and acoustic telemetry experimental design

Twenty brown meagres were collected at two different areas of the RNMCB: within the TPA (Cap Rédéris) and within the PPA (Cap Abeille) (Table 1, Fig. 1). Sampling was performed at three different periods (June–July 2016, October 2016 and June–August 2017), using dip nets (CAPERLAN, diameter 50 cm, mesh 3 mm), during the night and at a maximum depth of 10 m^50,55^. The individuals were brought slowly to the surface to avoid decompression injuries and stress. Once in the boat, the individuals were transferred to an anesthesia tank of 100 l, filled with in situ seawater and 15 ml of anesthetic solution (70% alcohol and 30% clove oil). Fish were measured, placed inverted, and tagged with V13P-1H acoustic transmitters (VEMCO, Nova Scotia, Canada, dimensions: 45 mm long by 13 mm in diameter). Transmitters were equipped with a pressure sensor and were programmed to emit signals every ca. 2 min. Only individuals over 30 cm total length were tagged in order to respect the welfare of the fish, since the weight of the transmitter should not exceed 2.6% of the fish weight^37^. The transmitters were placed in the coelomic cavity of the fish after a standard surgical procedure^55,56^. Tagging was conducted by trained and licensed scientists working under the authority and approval of the *certificat d’experimenter sur les animaux vertébrés vivants* (experimental live animal certificate) number 66.0801 (Elisabeth Faliex) of the CEFREM, University of Perpignan. The departmental council of the Pyrénées Orientales and the scientific council of the *Réserve Naturelle Marine de Cerbère-Banyuls* have granted us the authorizations to capture, mark and release the brown meagre individuals within this marine protected area. The manipulation of live vertebrate animals was also approved by the Ethic Committee on Animal Experimentation of the University of Barcelona (*Comitè d’Ètica d’Experimentació Animal de la Universitat de Barcelona*) and accredited by the Government of Catalonia (*Generalitat de Catalunya, Departament de Territori i Sostenibilitat, Direcció General de Polítiques Ambientals i Medi Natural*), under the licence no 11218, granted to Bernat Hereu. All operations were performed ensuring the minimum stress of the fish and were in compliance with the regulations expressed in the aforementioned licence and the ARRIVE guidelines^57^. The brown meagre is an iteroparous and gonochoristic species, but due to the lack of sexual dimorphism^58^, the sex of the individuals could not be determined. However, all individuals were bigger than the reported size at first maturity (20–30 cm^34,58^, indicating that they were all mature adults. The individuals were released at the same place of capture and followed by divers to monitor their swimming behavior and confirm their welfare. A fixed network of 17 submerged acoustic receivers (VR2W, VEMCO, Nova Scotia, Canada) was used to detect and record the movements of the brown meagre individuals within the RNMCB. Nine receivers were placed inside the TPA and the remaining in the PPA. The receivers had a detection range of about 200 m radius, depending on environmental conditions (e.g. turbidity, temperature, current and ambient noise), and they were recovered and changed every 6 months to recharge and recover the data.

**Table 1:** Summary of the sampling and acoustic detection information of the studied fish (non-averaged data).

Fish ID	Total length (cm)	Capture site	Tagging date	Tracking period (days)	Days detected	Residence index	Total detections	Detection/day
419	33	Cap Abeille	08/06/2016	504	364	0.72	39,904	79
424	37		08/06/2016	379	316	0.83	19,030	50
429	39		08/06/2016	367	229	0.62	12,350	34
3039	56		11/07/2016	482	279	0.58	39,932	83
3031	36		16/06/2017	506	497	0.98	57,262	113
3038	34		16/06/2017	506	492	0.97	43,683	86
3037	35		20/07/2017	365	339	0.93	66,705	183
455	37		21/07/2017	506	504	1	63,186	125
456	38		21/07/2017	21	21	1	2150	102
462	45		22/08/2017	100	59	0.59	2623	26
3032	35	Cap Réderis	04/10/2016	373	371	0.99	105,739	286
3033	35		04/10/2016	506	488	0.96	169,896	336
3034	33		04/10/2016	360	355	0.99	36,868	10
3035	32		04/10/2016	11	11	1	2415	220
3036	46		21/06/2017	506	505	1	43,946	87
459	36		21/06/2017	506	460	0.91	45,532	90
457	42		03/08/2017	506	506	1	51,455	102
458	47		03/08/2017	506	506	1	164,591	325
460	43		03/08/2017	506	505	1	66,404	131
461	55		03/08/2017	506	506	1	70,416	139

Residence index represents the proportion of days that each individual remained within the detection array.

*Fish ID* fish identification number.

### Data analysis

Acoustic telemetry data were retrieved from the receivers and gathered in a single integrated database by the VUE software (VEMCO, Nova Scotia, Canada). The depth of each fish was then averaged by hour, in order to homogenize the data over time. The residence index was calculated for each individual as the proportion of days at which individuals were detected in relation to the total number of days monitored^36^. The time of day (day/night) was determined according to the local sunrise and sunset times based on solar ephemeris calculation methods^19^.

Seawater temperature data were provided by the MPA managers recovered from several thermometers placed within the MPA (8 thermometers placed between 5 and 40 m depth in intervals of 5 m). The depth of the thermocline, when present, was calculated using a four-parameter nonlinear regression, fitted to the vertical profile of temperature^19,59^. Using the output of the model, the temperature was estimated for every 0.1 m depth and the mid-depth of the thermocline was defined as the point at which the first derivative of the model corresponded to the fastest range of temperature change. Thermocline depth was only calculated for the profiles where the total temperature difference between the surface and the deepest measures was higher than 3 °C^19^.

In order to uncover general patterns of depth segregation among individuals and over time, fish depth was analyzed in a standardized and non-dimensional form (i.e. zero mean and unit variation), calculated with the “scale” function of the base R package. A cluster analysis was performed on the total averaged Z-scores per fish using the “factoextra” R package^60^, to identify distinct groups of fish that presented different overall mean of standardized depth (SS: shallow/shallow and DS: deep/shallow). The standard scores (or Z-scores) were then averaged by month for each fish and two intra-annual periods (warm and cold) were identified based on the data of the DS group: months with at least 3 fish showing positive mean Z-scores were included in the warm period. Differences of mean depth between individuals, groups, and periods were assessed by ANOVA or Kruskal–Wallis tests (when parametric assumptions were not verified).

To detect significant seasonal and nyctemeral variation of fish depth according to their group, as well as the effect of collection site and fish size, a linear mixed-effect model (LMEM) was applied to the log-transformed (ln (x + 1)) fish depth data^61^. The fish identification number (ID) was included as a random effect, while collection site, fish size, and interaction of the period, day/night time, and group were included as fixed factors. The collection site was used to test the effect of the MPA protection level, assuming that each individual remained at the same MPA area since the brown meagre is recognized as a sedentary species that lives within a small home range area (< 1km^2^^36^). The year was not included in the model because different fish were tagged in different years. To cope with heterogeneity, a variance structure was added to the model, which assumed heterogeneity of variances per fish (*i*) but homogeneity within fish over temperature (*j*). The significant effect of each fixed factor was obtained by likelihood-ratio tests of the full model against the model without the effect in question. The final model was selected according to Akaike information criterion (AIC). Generalized additive mixed models (GAMM) were used to assess the effect of temperature and thermocline depth on fish depth (*Depth*) since they are more flexible than parametric models and let non-linear patterns appear in the data^61^. The model was fitted to the log-transformed fish depth (ln (x + 1)), using the entire data set, to assess the effect of temperature (fixed effect), with one intercept (α) and fish ID as a random effect (*a*_i_) with different variances ($\sigma _{i}^{2}$), as in Eq. (1).

1 $$Depth_{{ij}} = ~\alpha + a_{i} + f\left( {Temp_{j} } \right) + \varepsilon _{{ij}} \quad \varepsilon _{{ij}} \sim N\left( {0,\sigma _{i}^{2} } \right).$$

To assess the effect of thermocline depth (*Thermo*) on the fish depth, a second generalized additive mixed model was fitted to the log-transformed fish depth (ln (x + 1)), using only data when thermocline was detected. As in the previous model, a random effect (fish ID, *a*_i_) was also included in the model, following Eq. (2).

2 $$Depth_{{ij}} = ~\alpha + a_{i} + f\left( {Thermo_{j} } \right) + \varepsilon _{{ij}} \quad ~\varepsilon _{{ij}} \sim N\left( {0,\sigma _{i}^{2} } \right).$$

The variance of depth per day/night time was used as a proxy of fish activity (*Activity*), assuming that higher variance reflected bigger ranges of vertical movements, and therefore, higher activity levels^19^. A linear mixed model was fitted to the log-transformed variance of fish depth (ln (x + 1)), in order to uncover significant variation of fish activity over time, including the interaction of day/night time and fish group, as well as the collection site and fish size as fixed effects and fish ID as a random effect. The significant effect of each fixed factor was obtained as described above. Effect of temperature (fixed effect) was tested by a generalized additive mixed model (GAMM), following the Eq. (3), fitted to all data of the log-transformed variance (ln (x + 1)), including fish ID as a random effect:

3 $$Activity_{{ij}} = ~\alpha + a_{i} + f\left( {Temp_{j} } \right) + \varepsilon _{{ij}} \quad \varepsilon _{{ij}} \sim N\left( {0,\sigma _{i}^{2} } \right).$$

The effect of thermocline depth (*Thermo*) on fish activity (log-transformed) was also tested by a GAMM (Eq. (4)), using only data when thermocline was detected and including fish ID as a random effect:

4 $$Activity_{{ij}} = ~\alpha + a_{i} + f\left( {Thermo_{j} } \right) + \varepsilon _{{ij}} \quad \varepsilon _{{ij}} \sim N\left( {0,\sigma _{i}^{2} } \right).$$

The performance of all models was visually evaluated by the inspection of fitted vs residuals and fitted vs fixed factors values. The models were fitted using the functions “lme” from the R package “nlme”^62^ and “gamm” from the R package “mgcv”^63^. All data treatment was performed in R software^64^.

## Results

Among the 20 fish tagged in this study, three (fish no 456, 462, and 3035) were not included in the analysis, since they disappeared from the study site few days after release (Table 1). The remaining individuals stayed within the detection array and were monitored over long tracking periods (minimum of 359 days). The analyzed fish showed a high residence index (minimum of 58%, Table 1), with 13 out of 17 fish spending more than 90% of the monitoring period within the RNMCB**.**

**Figure 2:**
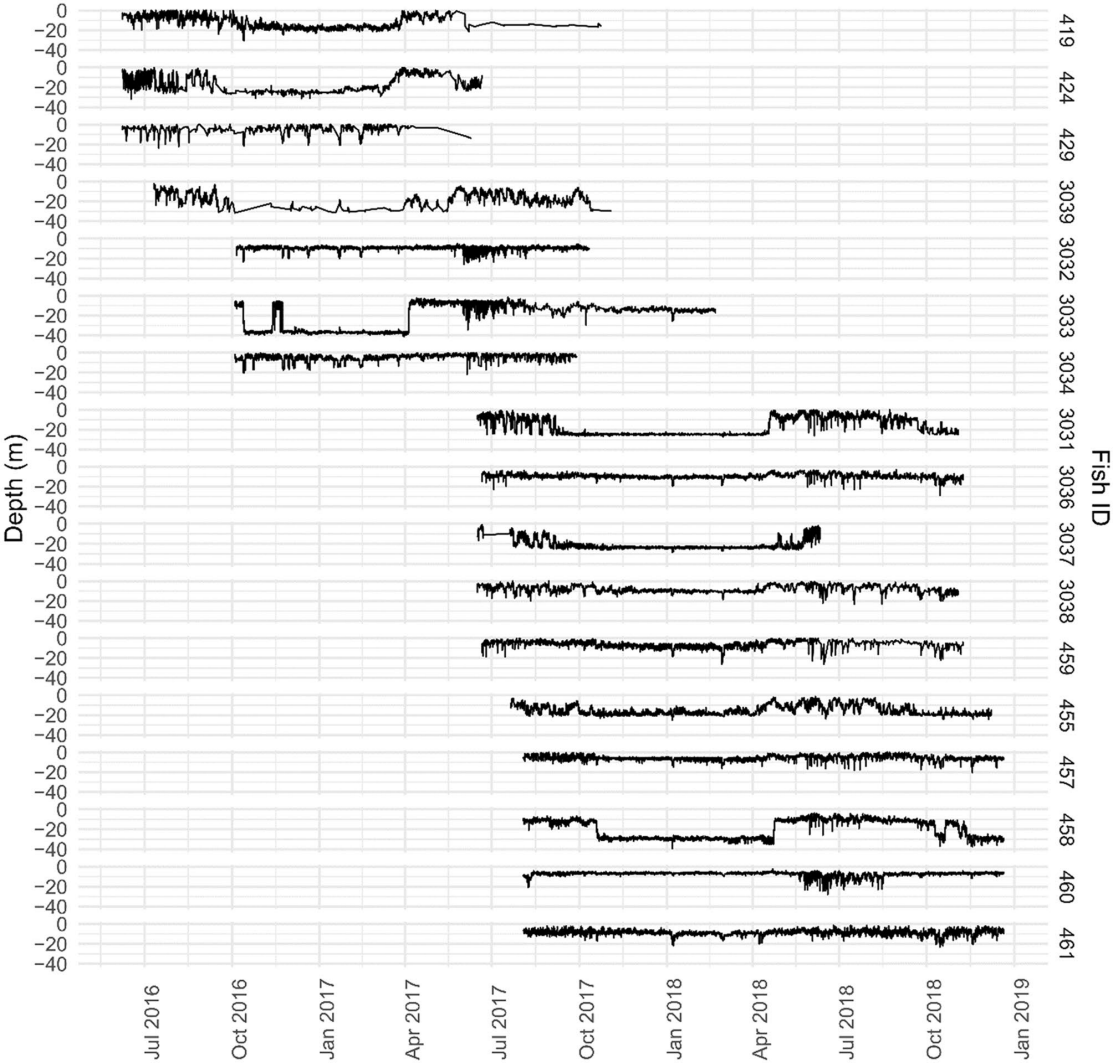
Individual depth during the study period. Mean depth per hour of each fish ID (identification number) over time.

The depth of the analyzed fish varied between surface waters to a maximum registered at 41.8 m (Fig. 2). Some fish revealed intra-annual variability in their depth, occupying different layers of the water column at different times of the year.

**Figure 3:**
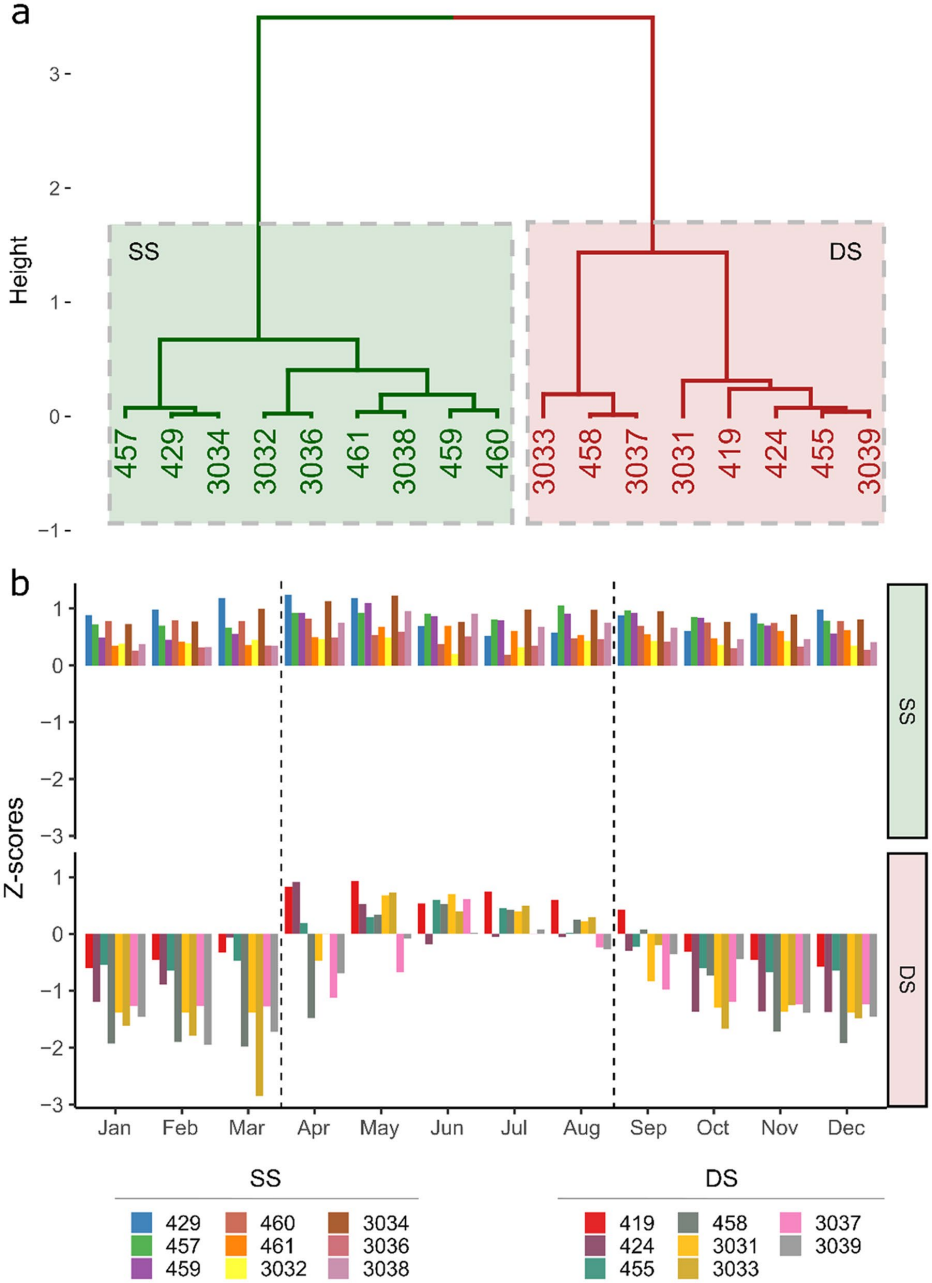
Definition of fish groups and periods. (**a**) Clustering analysis on the total mean fish depth Z-scores, identifying two fish groups (*SS* shallow/shallow, *DS* deep/shallow) and (**b**) monthly mean of fish depth Z-scores (all years included) to define two periods, delimited by dashed lines (Warm: April to August and Cold: January to March and September to December). Numbers represent the fish identification number.

**Figure 4:**
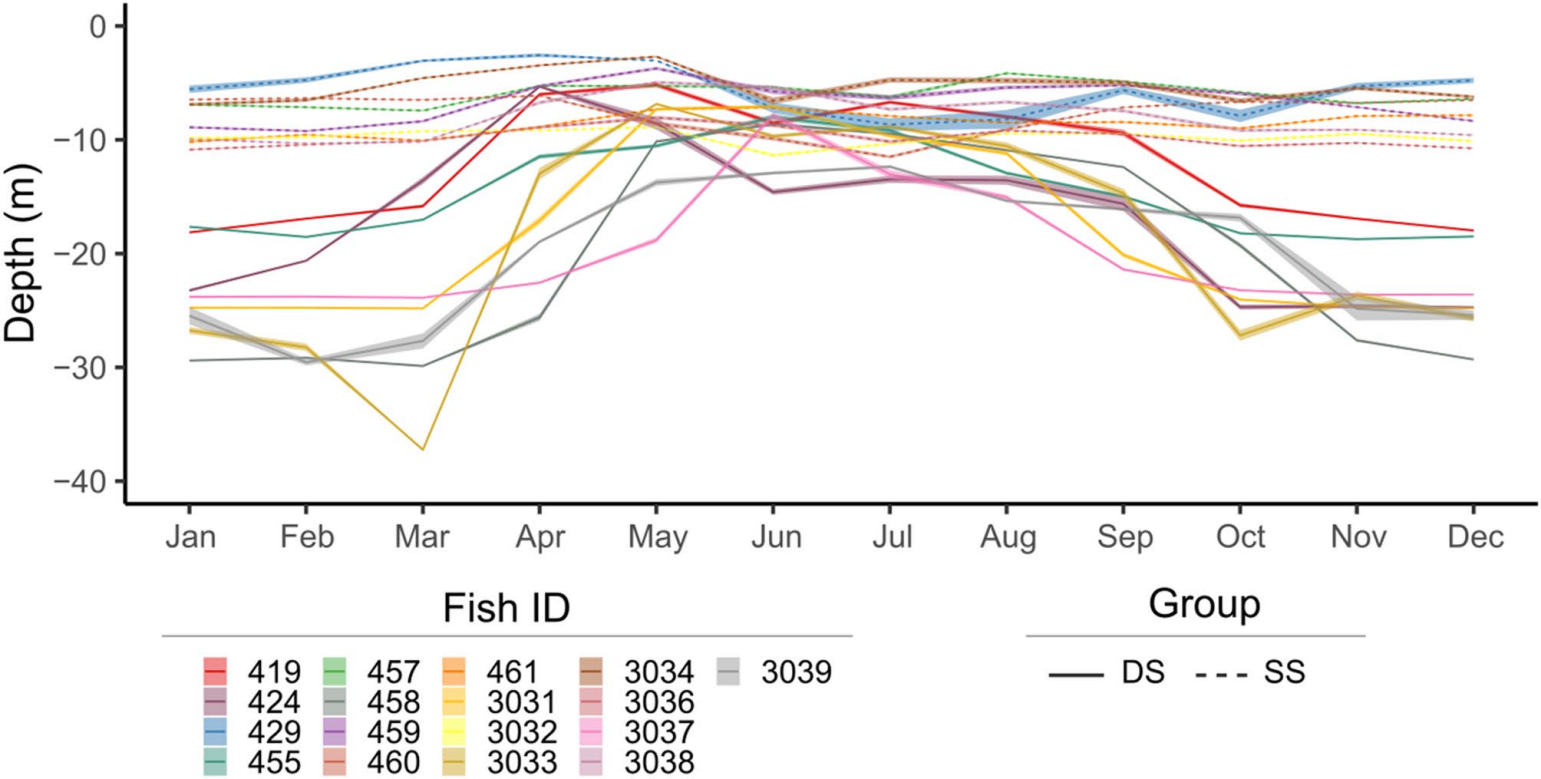
Monthly individual depth. Mean (± SE) depth of each fish identified by their identification number (Fish ID) and by group (*SS* shallow/shallow, *DS* deep/shallow), over the study period (all years included).

The intra-annual variability of fish depth appeared to be individual dependent (Fig. 3). This variability allowed us to identify two fish groups (SS: shallow/shallow and DS: deep/shallow) and two periods (warm and cold), based on the total and monthly mean Z-scores of fish depth over time. The SS group included the fish that showed depths above the annual population mean (0 standard score) during all year round. The DS group was composed of the fish that showed a transition from deeper waters during the cold period (September to March) to shallow waters during the warm period (from April to August). The SS group included 47% of the individuals and presented an overall mean depth of 8.02 ± 0.01 and 7.53 ± 0.03 during the cold and warm periods, respectively, while the DS group (53% of the individuals) changed from 22.5 ± 0.04 m during the cold periods to 12 ± 0.05 m during the warm period. The overall mean depth between and among groups and periods were significantly different (two-way ANOVA, p-value < 0.001). During the cold period, a stratification of depth among individuals of the DS group was evident, with a significant difference of monthly mean depth between individuals (Kruskal–Wallis test, p-value < 0.001), with values ranging from 27.2 ± 0.5 m to 37.2 ± 0.03 m (Fig. 4).

**Figure 5:**
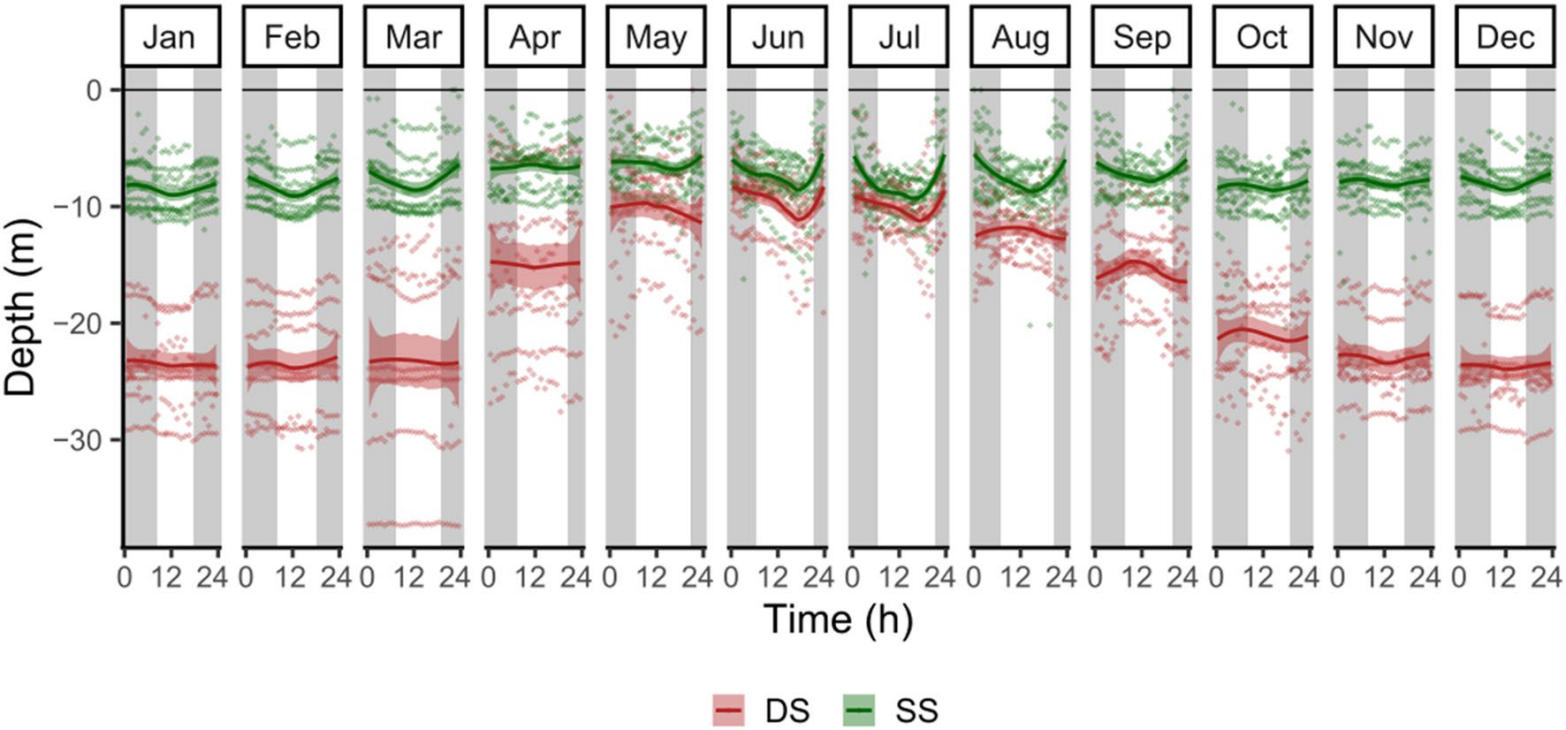
Variation pattern of daily and seasonal vertical movements. Each point is the hourly mean depth of each fish, identified by group (*SS* shallow/shallow, *DS* deep/shallow). Lines represent Locally Weighted Least Squares Regression (LOESS) fit model. White and grey areas represent day and night periods, respectively.

The individuals of *S. umbra* appeared also to vary their depth between day and night with a general pattern of increasing depth during the day (Fig. 5). In June and July, the vertical movement of both groups was evident, remaining at shallow waters during the night and diving into deeper waters especially just before sunset. In August and September, the pattern of vertical movements contrasted between groups, with the SS fish increasing their depth during the day, while the DS fish showed the opposite vertical movements. The results from the linear mixed-effects model (LMEM) confirmed the significant seasonality and daily patterns of depth, which are dependent on the group (expressed by the significant effect of the interaction between group, period and day/night time, LMEM, F = 15,362, p-value ≤ 0.001). This temporal variation of fish depth though, was not affected by the collection site or fish size (LMEM, F = 1.72, p-value = 0.19 and F = 1.97, p-value = 0.16, respectively). The final model (Table 2), confirmed the significant overall higher depth of the DS fish, especially during the cold period, as well as during night time. Differences between day/night time appeared to be dependent on the group but not on the period (LMEM, p-value = 0.17, Table 2), confirming the general utilization of shallower waters during the night over the whole study period.

**Table 2:** Results of the linear mixed effects model testing the effect of period (warm and cold), day/night time (DN), and group (*SS* shallow/shallow, *DS* deep/shallow) on the fish depth.

	Estimate	Std. error	df	t value	p-value
(Intercept)	3.049	0.080	107,804	38.198	< 0.001
Period warm	− 0.602	0.005	107,804	− 129.581	< 0.001
DN night	0.018	0.004	107,804	4.151	< 0.001
Group SS	− 0.928	0.110	15	− 8.460	< 0.001
Period warm: DN night	0.010	0.007	107,804	1.368	0.171
Period warm: group SS	0.541	0.006	107,804	96.541	< 0.001
DN night: group SS	− 0.108	0.005	107,804	− 21.418	< 0.001
Period warm: DN night: group SS	− 0.021	0.008	107,804	− 2.551	0.011

**Figure 6:**
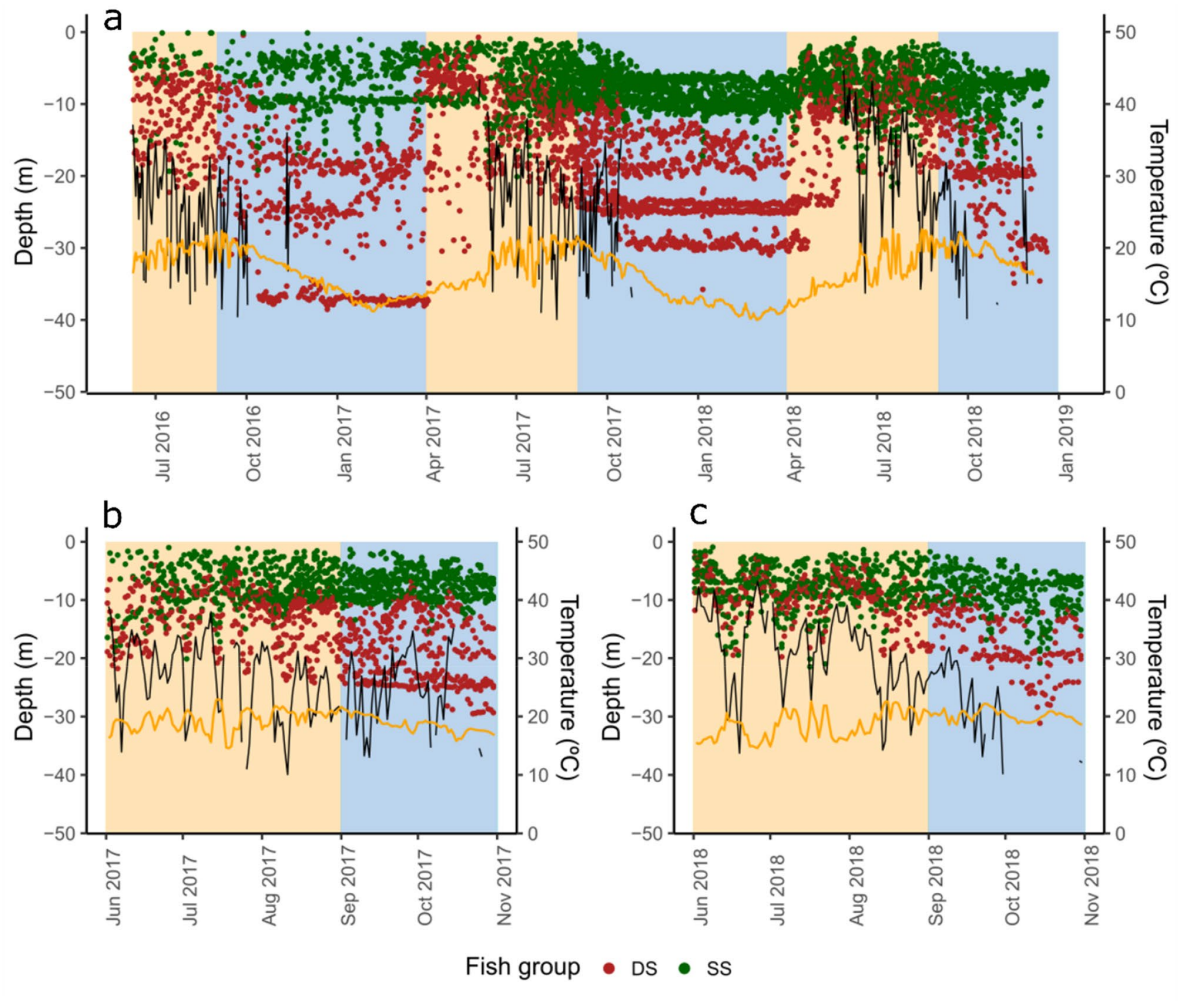
Fish depth over the study period. Points represent the daily mean depth of all fish identified by group (*SS* shallow/shallow in green, *DS* deep/shallow in red), over the whole study period (**a**) and a subset of data when the thermocline is present in 2017 (**b**) and 2018 (**c**). Black lines represent thermocline depth and orange lines represent temperature (°C) at 15 m depth. Background colors represent cold and warm periods in blue and light orange, respectively.

**Figure 7:**
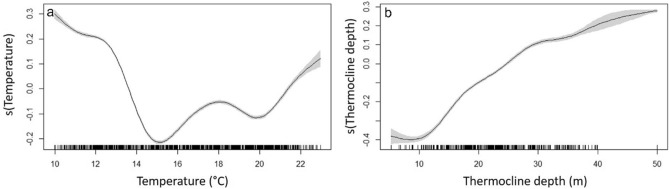
Results of the GAMM models on the fish depth during the study period. Effect of seawater temperature (at 15 m depth) (**a**) and thermocline depth (**b**). Lines represent the estimated smoothing curves from each model and shaded areas represent the 95% confidence interval for the mean shape of the effect.

The temperature at 15 m depth varied seasonally over the study period (Fig. 6), with higher values (monthly mean > 20 °C) between August and September and minimum values (monthly mean < 12 °C) in February and March. The temporal variability of fish depth appeared to be significantly affected by temperature (GAMM, F = 1527, p-value < 0.001), indicating that fish decrease their depths at temperatures between 15 and 20 °C, but return to deeper waters at both colder and warmer temperatures (Fig. 7a). The thermocline was only detected between June and November, with a range between 5.2 and 50 m depth (Fig. 6). When present, the thermocline positively affected the depth of the fish (GAMM, F = 1359, p-value < 0.001), and appeared to limit the maximum depth of the fish, in particular between June and August (Figs. 6, 7b).

### Fish activity

**Figure 8:**
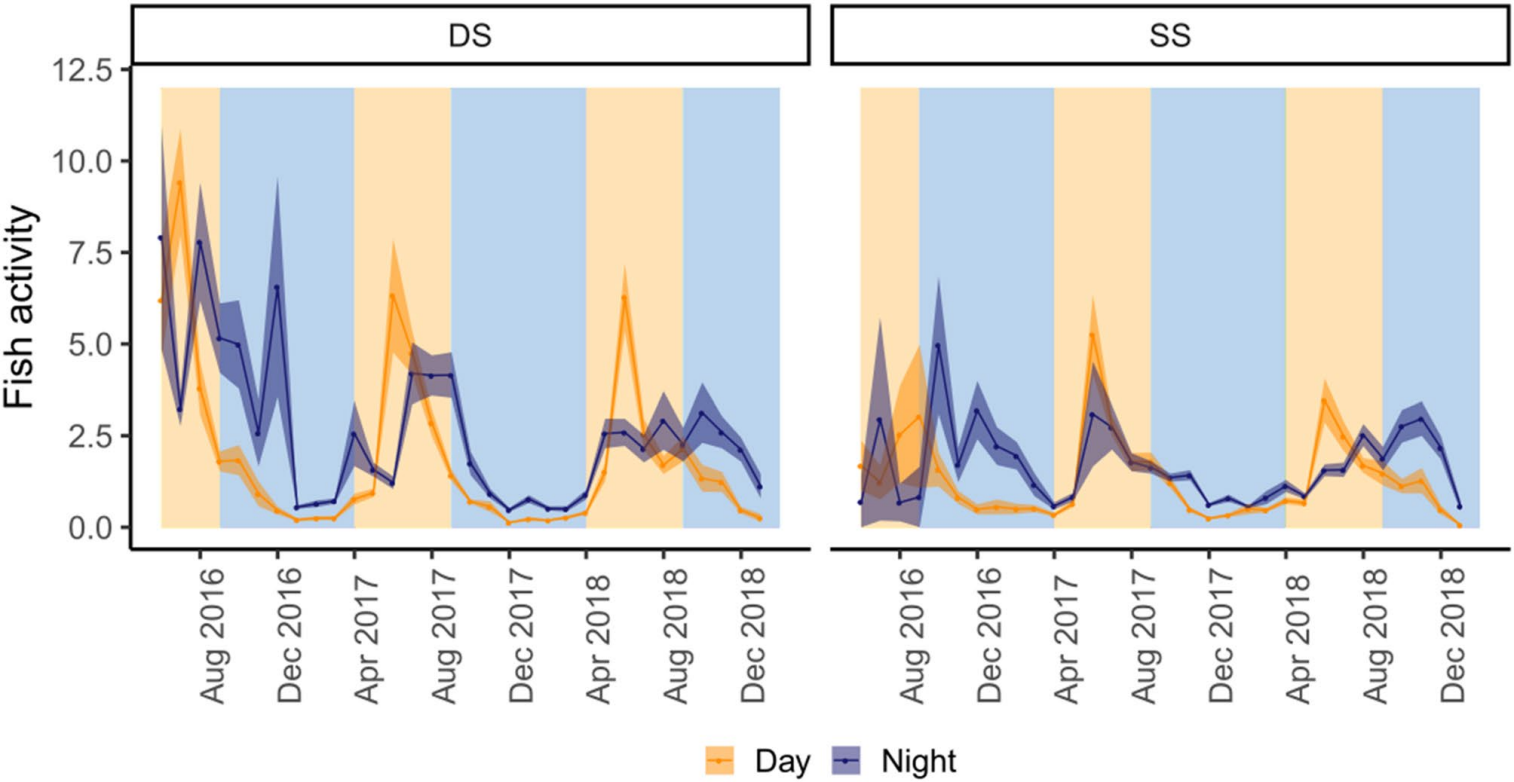
Monthly mean fish activity of each group per day and night, over the study period. *SS* shallow/shallow, *DS* deep/shallow. Background colors represent cold and warm periods in blue and light orange, respectively.

The activity of the fish varied over time with conspicuous peaks of night activity during the cold period and of day activity during warm periods (Fig. 8). Differences in activity between day and night times were confirmed by the model (Table 3) and this temporal pattern appeared to be similar for both groups (LMEM, p-value = 0.984). Fish were significantly more active during the warm periods, but this pattern differed between groups, with the DS group showing higher discrepancy between periods than the SS group (Fig. 8, Table 3).

**Table 3:** Results of the linear mixed effects model testing the effect of period (warm and cold), day/night time (DN), and group (*SS* shallow/shallow, *DS* deep/shallow) on the vertical activity of the fish (variance of depth per each day/night time).

	Estimate	Std. error	df	t value	p-value
(Intercept)	0.147	0.016	20	9.040	< 0.001
Period warm	0.255	0.010	13,540	24.349	< 0.001
DN night	0.115	0.009	13,530	12.167	< 0.001
Group SS	0.009	0.022	20	0.414	0.683
Period warm: DN night	− 0.090	0.015	13,530	− 6.136	< 0.001
Period warm: group SS	− 0.105	0.014	13,540	− 7.345	< 0.001
DN night: group SS	0.000	0.013	13,530	− 0.033	0.974
Period warm: DN night: group SS	− 0.032	0.020	13,530	− 1.608	0.108

**Figure 9:**
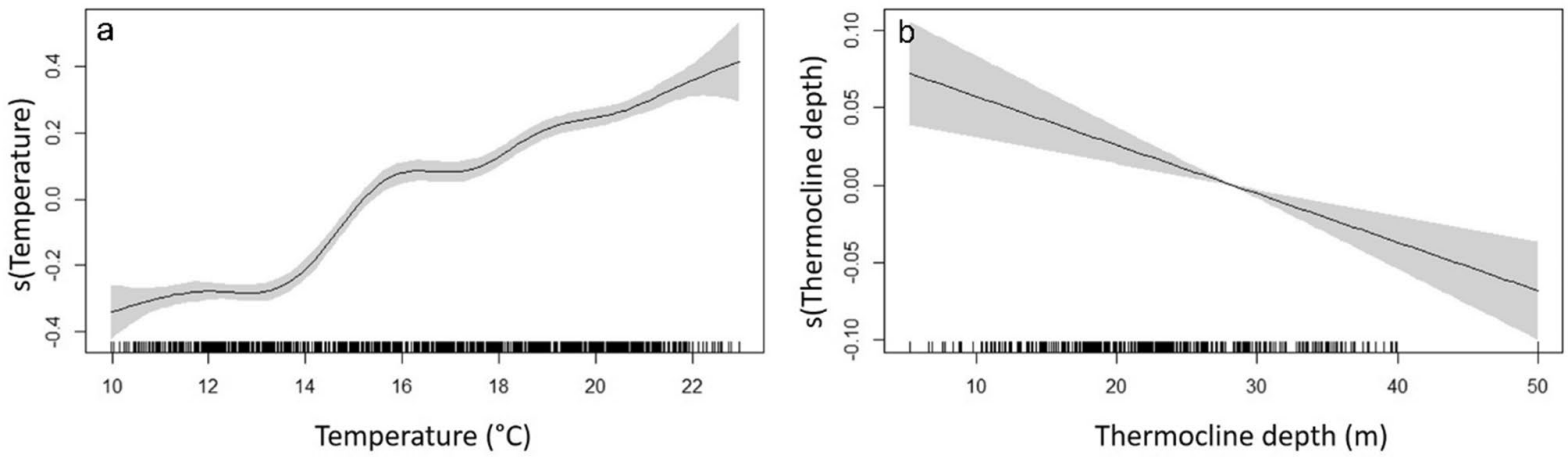
Results of the GAMM models on the fish activity during the study period. Effect of seawater temperature (at 15 m depth) (**a**) and thermocline depth (**b**). Lines represent the estimated smoothing curves from each model and shaded areas represent the 95% confidence interval for the mean shape of the effect.

Like for depth, fish activity was not affected either by the collection site or fish size (LMEM, F = 0.09, p-value = 0.77 and χ^2^ = 1.02, p-value = 0.31, respectively). Fish activity was significantly positively affected by temperature and negatively affected, when present, by the depth of the thermocline (GAMM, t-value = 209.7, p-value < 0.001, t-value = 18.79, p-value < 0.001, respectively, Fig. 9).

## Discussion

The acoustic telemetry system used in this study provided valuable information regarding the behaviour of the brown meagre, *Sciaena umbra*, in one of the oldest MPAs in the Mediterranean Sea (the *Réserve Naturelle Marine de Cerbère-Banyuls*, RNMCB), increasing our understanding of the ecology of this species. This technique provided detailed information on the fish vertical movements over a long time period, confirming their high site fidelity (most of the individuals remained within the MPA more than 90% of the monitoring time), as suggested in previous studies^36^. Furthermore, the results allowed to uncover seasonal and nyctemeral patterns, that show not only individual-based behaviour (foraging and reproductive) but also population-based behaviour (segregation or aggregation of individuals).

### Seasonal variability in depth uses

The population of brown meagre, at least in our study site, was composed of two main groups of individuals showing different seasonal patterns of depth use: the individuals that live constantly in shallow water (the SS group) and the individuals that shift from deeper waters (up to 50 m depth) during the cold months to shallow waters during the warmer months (the DS group), when all individuals gathered close to the surface.

Individual segregation (or aggregation) might be generally included in two main types: habitat segregation and social segregation^65^. Habitat segregation refers to the use of different physical environments, influenced by trophic factors, habitat type, vulnerability to predation, and abiotic environmental conditions. Social segregation is the tendency for a species to form groups, for instance, by age, sex, and/or size^65–67^.

During the cold period, our results revealed a stratified distribution of *S. umbra* individuals through different depth ranges, in particular within the DS group. The cold period represents the time when the brown meager shows higher foraging activity^32^ and, therefore, the observed segregation likely reduces the intra-specific competition for food and space, such as previously suggested for other species^68^. In the warm period, the aggregation of *S. umbra* individuals at shallow waters is likely a reflection of social aggregation, stimulated by their reproductive activity.

In our study, the ecological drivers that explain the separation of the individuals in SS and DS groups, are difficult to unveil and confirm. Size and age may affect the depth distribution of fish, often leading to, for instance, an aggregation of individuals of the same age^66,67^. However, these factors have probably little effect on the vertical distribution of the brown meagre in our study since all individuals were mature adults and no significant effect of size was shown in our results. The two contrasting patterns could also reflect the effect of human activities on the behaviour of the brown meagre collected at different areas of the MPA. For instance, boat engine noise has been already shown to significantly affect the behaviour of various fish species, including the brown meagre^69–72^, with significant effects on fish vertical movements^69^. Here, the effect of human activities was tested by the influence of the MPA protection level, assuming that fish collected within the totally protected area (where all human activities, including boat traffic, are restricted) are under lower anthropogenic pressures than those collected within the partially protected area (where boat traffic is allowed, especially linked to the high level of diving activity during summer period). Our results indicate that this factor did not influence the observed fish segregation pattern, rejecting this hypothesis. Still, since both collection sites were under some level of protection, the significant impact of anthropogenic activities on brown meagre behaviour cannot be excluded in areas outside the MPA. A possible explanation for the two distinctive intra-annual patterns of fish depth observed in our study is sex-dependent depth distribution. This hypothesis could not be confirmed in our study and this type of sexual-dependent contrasting behaviour has never been reported, so far, for *S. umbra*. However, sex-dependent segregation is known for many species of teleosts and elasmobranchs^65^. For instance, the male and female dolphinfish (*Coryphaena hippurus*) have different dietary preferences, which affect their foraging behaviour and the associated habitat preference^68^. Although this might be possible for *S. umbra*, to our knowledge, sex-dependent diet has never been reported for this species. The speculative sex-dependent depth segregation of the brown meagre could, therefore, be linked to their reproductive behaviour, as reported for other species. For instance, male cod (*Gadus morhua*) dominate the spawning aggregation sites and females migrate in and out of these areas during the reproduction season^73^. If this was true for the brown meagre, it was possible that males represent the SS group, inhabiting caves and protected shelters^22–24^, which, in RNMCB, are more common in the shallow rocky areas. During the spawning season, males could be visited by females that would migrate from deeper waters, probably attracted by their spawning calls^53^. Indeed, our results reveal that the two groups gathered during the typical reproduction season reported for the brown meagre, which is recognized to form breeding aggregations between May and August^34,53,58,74^. If the hypothesis of sex-dependent group behaviour was true, it is evident that both sexes would have to aggregate for reproduction purposes. Still, although possible, the sex-dependent group behaviour hypothesis would require further confirmation.

The reproduction of the brown meagre occurs during the warmer months which represent the season with better conditions for the survival of offsprings^58^. Our results show a significant effect of temperature on the intra-annual pattern of the vertical distribution of *S. umbra*, which matches with the reported spawning season and highlights the potential role of temperature in controlling the timing and success of their reproduction. In addition, we further uncover the importance of the thermocline, which appeared to control the maximum depth of the brown meagre between June and August. Similar suprathermoclinal depth selection was also reported for the common dentex, *Dentex dentex*^19^, and for the Pacific bluefin tuna, *Thunnis orientalis*^75^. However, these authors have hypothesized that the effect of the thermocline in the fish thermal niche selection is likely related to their foraging activity, whether by increasing their physiological capacity for predation (the effect of temperature on fish swimming speed^19^, or indirectly by increasing food availability^75^. The brown meagre decrease their feeding activity during the reproduction season^34^ and therefore, we consider that these hypotheses are less likely for this species. We believe that this preference for suprathermoclinal hot water during the peak of the active reproductive season might be related to a physiological optimization strategy^76,77^, in this case, to increase reproductive success. The spawning activity of the brown meagre is triggered when temperatures exceed 18 °C^34^, the approximate temperature at which the thermocline occurs in our study site. This suggests that the thermocline might represent the depth limit at which fish have to remain to attain a successful reproduction by increasing, for instance, egg quality^78^ and/or the probability of egg survival^79,80^. In the light of the expected extension of summer conditions in the Mediterranean Sea^81^, it is possible that the reproductive behaviour of this species might be affected by the forecasted climate change. Its potential effects on the reproductive efficiency are still unknown with possible positive or negative consequences on, for instance, sexual aggregation synchronization, gonadal maturation, fish fecundity, egg quality, and survival, which, ultimately controls the population productivity^17,82^. This stresses the need for further behavioural and physiological studies that can be integrated into ecological models. This would improve our knowledge of the future response of this species to climate change and provide insights for its management and conservation^83^.

### Nyctemeral variability in depth uses

At the scale of the 24 h cycle, all individuals used deeper layers of the water column during the day and shallower layers during the night, which is probably linked to their foraging activity. The diet of this species is composed mainly of crustaceans^34,84,85^ but fish prey becomes more important in the diet of adult individuals^34^. In this study, only *S. umbra* adults were monitored and, therefore, it is possible that the use of shallower waters indicated an active search for pelagic fish prey rather than benthic crustaceans. The nocturnal foraging behaviour might be also perceived in activity results, which indicate an overall higher activity of the fish during the night time, regardless of the group. According to previous studies, the Sciaenidae family and, in particular, the brown meagre, have relatively large otoliths^86^, which has been suggested as an adaptive strategy for acoustic communication and indicates a more active behaviour during the night^31,70,87^. Our results are in agreement with the aforementioned studies, but they further suggest different day/night activities depending on the time of the year. Here, we show that during the warm period (in particular in June and July), the activity is higher during the day than the night. This is likely the reflection of the conspicuous peak in depth variation of the brown meagre at dusk, which might be associated with the spawning behaviour of this species. At dusk, the brown meagre emit sounds associated with the courtship behaviour, indicating a peak of reproductive activity during this particular time of day^86,88^. Just before courtship, males of sciaenid family remain calm close to the bottom, emitting sounds. When one female is receptive, both individuals rise towards the surface, where gametes are released^86^, which matches the peak of depth variation observed in our results. This has particular relevance since it suggests that the dusk hours, especially in June and July, are of paramount importance for the reproductive success of the brown meagre and should be considered as periods of high vulnerability, with interest for the protection of the species within the MPA.

### Protection and management implications

The brown meagre is an endangered species and very vulnerable to spearfishing and recreational fishing activities due to their indolent behaviour and accessibility^23,24^. This is why MPAs and fishing restrictions are critical for their protection, which promotes the recovery of their populations^31^. However, we advocate that further preservation measures are required during critical moments of their life cycle, such as social spawning aggregations. Our results suggest that the peak of the brown meagre aggregations in the RNMCB occur mainly in shallow waters, during the dusk hours between June and July, presumably associated with their breeding activity. Although this reproductive timing should be confirmed in other MPAs, we believe that this information should be considered when developing mitigation strategies for the protection of *S. umbra*. This study provides new insights into the potential effect of human activities that might indirectly affect the population of the brown meagre. Indeed, not only the direct impact of the fishing activity on their abundance but other pressures, like diving and boat traffic, might reduce the reproductive success of this species and ultimately reduce their abundance. Boat noise is likely to affect the acoustic communication between *S. umbra* individuals^70,71^, which are of paramount importance during the spawning season^86,88^. Likewise, diving might lead to a modification of their behaviour, potentially preventing the aggregation of individuals^89^.

This is all the more important for an endangered species in the process of recolonization, as is the case in several marine protected areas in the Mediterranean. Reproductive success is complementary to the protective effect for adult populations. A modeling study shows that protection is all the more effective as the home range of the species is reduced and the size of the reserve is significant^90^.

Although further studies should be performed to quantify the effect of these human activities on the behaviour of the brown meagre, we advocate that diving and boat traffic restrictions should be implemented within MPAs during these critical periods, which would certainly contribute to the protection and recovery of the populations of the brown meagre within and possibly outside MPAs.

## Conclusion

Overall, this study uncovers previously unnoticedvariability in the bathymetric distribution of brown meagre at different time scales, which are likely associated with their trophic (during cold months) and reproductive (during warm months) activities. Furthermore, we identify where and when vulnerable periods of its life cycle occur, in shallow waters, in particular the dusk hours in June and July, when the spawning activity of the brown meagre appear to be in its maximum. Such information is of great importance to improve the protection measures for this species and to mitigate the impact of human activities that might disturb their behaviour during these periods. Preliminary results on their spatial distribution confirmed the previously reported high site fidelity^36^: most of the individuals remained within the MPA more than 90% of the monitoring time, they occupy small home ranges (< 1 km^2^), and they reside nearby their collection site (Marques et al., unpublished data). However, this study was only focused on the little known bathymetric distribution of the brown meagre and, therefore, further detailed investigations on the horizontal distribution of this species should be performed to uncover finer spatial movements within the RNMCB. Likewise, complementary studies addressing movements of *S. umbra* (and other endangered species) in both dimensions would be of great value to better understand their ecology and assist to define better management actions to protection them and help their future population recovery in other MPAs.

## Data Availability

The datasets generated and analysed during the current study are available from the corresponding author on reasonable request.
